# Semi-CAM: A semi-supervised deconvolution method for bulk transcriptomic data with partial marker gene information

**DOI:** 10.1038/s41598-020-62330-2

**Published:** 2020-03-25

**Authors:** Li Dong, Avinash Kollipara, Toni Darville, Fei Zou, Xiaojing Zheng

**Affiliations:** 1 Department of Biostatistics, University of North Carolina at Chapel Hill, Chapel Hill, North Carolina USA; 2 Department of Pediatrics, University of North Carolina at Chapel Hill, Chapel Hill, North Carolina USA

**Keywords:** Statistical methods, Predictive markers

## Abstract

Deconvolution of bulk transcriptomics data from mixed cell populations is vital to identify the cellular mechanism of complex diseases. Existing deconvolution approaches can be divided into two major groups: supervised and unsupervised methods. Supervised deconvolution methods use cell type-specific prior information including cell proportions, reference cell type-specific gene signatures, or marker genes for each cell type, which may not be available in practice. Unsupervised methods, such as non-negative matrix factorization (NMF) and Convex Analysis of Mixtures (CAM), in contrast, completely disregard prior information and thus are not efficient for data with partial cell type-specific information. In this paper, we propose a semi-supervised deconvolution method, semi-CAM, that extends CAM by utilizing marker information from partial cell types. Analysis of simulation and two benchmark data have demonstrated that semi-CAM outperforms CAM by yielding more accurate cell proportion estimations when markers from partial/all cell types are available. In addition, when markers from all cell types are available, semi-CAM achieves better or similar accuracy compared to the supervised method using signature genes, CIBERSORT, and the marker-based supervised methods semi-NMF and DSA. Furthermore, analysis of human chlamydia-infection data with bulk expression profiles from six cell types and prior marker information of only three cell types suggests that semi-CAM achieves more accurate cell proportion estimations than CAM.

## Introduction

Bulk transcriptome profiles from tissue with a mixture of cell types distinct in their origins and functional states has hindered discovery of disease mechanisms. There is a critical need to decompose bulk expression data into cell type-specific expression and/or cell composition to better understand cell type-specific responses for medical research. For example, knowledge of global cell type-specific immune responses is critical for developing effective vaccines^1^; mouse models have suggested immune cell compositions reflect immune-related diseases^2^.

Over the last decade, traditional experimental tissue isolation methods such as laser-capture microdissection or fluorescence-activated cell sorting and single-cell technologies have been used to generate cell type-specific expression. Despite the power of these technologies, they may affect cell physiology and gene expression due to chemical and/or mechanical injury of the cells^3^ and are not readily practical on large sample cohorts. In contrast, bulk gene expression data are widely available in public datasets, such as NCBI GEO^4^ and UK Biobank^5^. Thus, there is a compelling need to develop statistical deconvolution approaches that can take full advantage of bulk transcriptomic data for estimating cell proportions and cell type specific expressions.

Existing statistical deconvolution approaches can be categorized into two major classes, unsupervised and supervised methods, based on their requirements for prior information. Unsupervised deconvolution methods do not require any prior information. The most commonly used algorithm for unsupervised deconvolution is Non-negative Matrix Factorization (NMF), which directly factorizes bulk gene expression into cell type-specific expression and cell proportions with “non-negative” and “sum-to-one” constraints^6–8^. Other unsupervised algorithms perform deconvolution analysis by first identifying marker genes. For instance, the convex geometry-based methods^9^ assume that the gene expression of a pure cell type is non-negative, and solve a classical convex optimization problem to determine the sources of cell types by finding the facets of the convex hull constructed by the observed bulk gene expression profiles. Convex Analysis of Mixtures (CAM)^10^ is another example. CAM first identifies novel marker genes by geometrically locating the vertices of the simplex that most tightly encloses the bulk gene expression profiles, and then estimates the cell proportions of the cell mixtures using the identified marker genes.

In contrast to the unsupervised methods, supervised approaches estimate cell proportions utilizing the following two types of prior information. First, the most commonly used prior information by the existing supervised deconvolution methods, such as DECONVOLUTE^11^, DeconRNASeq.^12^ and CIBERSORT^13^, is the reference cell type-specific gene expression signature. The accuracy of deconvolution is highly influenced by the variations between the bulk expression profiles and the reference cell type-specific gene expression signatures due to potential batch or other technical differences between the bulk and reference samples. PERT^14^ attempts to improve the deconvolution accuracy through accounting for these transcriptional variations by estimating a shared perturbation factor across all cell types. Nevertheless, these methods require reference cell type specific gene expression signatures from *all* cell types. ISOLATE^15^, DeMix^16^ and DeMixT^17^ relax this requirement and can allow one cell type to have missing reference gene expression signatures.

Second, some deconvolution methods require marker genes of each cell type as prior information. Ideal marker genes are those specifically and highly expressed on a single cell type but not on all the other cell types, and are resistant to the influence of environmental factors or treatment conditions. The Digital Sorting Algorithm (DSA)^18^, a marker-based deconvolution algorithm, uses only gene expressions from known cell type specific markers to infer cell type proportions and cell type-specific expression simultaneously. In contrast to DSA where only the expression levels from the cell type specific markers are used for deconvolution, the semi-supervised marker-based approach of Gaujoux and Seoighe^19^, Nonnegative Matrix Factorization (semi-NMF) uses the expression from all genes but adds constraints on the expression of the known marker genes similar to DSA to assure that those markers only express on one and only one cell type. At the meantime, for the rest of the genes, semi-NMF employs the Nonnegative Matrix Factorization estimation process for deconvolution. For situations that the marker genes are not available from each cell type, Becht *et al*.^20^ developed Microenvironment Cell Populations-counter (MCP-counter), which first searches for marker genes by integrating gene expression data from public databases, and then adopts identified markers for deconvolution. However, MCP-counter may miss those cell types not public available.

Unsupervised methods are conducted in a completely unsupervised fashion; they may decompose the mixture expressions into components that are not related to the actual cell types in the mixture^19^. Also, these methods completely disregard available information and thus are not efficient for data with partial cell type-specific information. Supervised deconvolution methods, in contrast, rely on prior information on cell proportions, or gene signatures, or marker genes of each cell type. Such information may not be readily available. For example, collecting cell proportions on all cell types can be difficult for most tissues. Even for easily accessible tissues, such as whole blood, getting neutrophil counts from stored samples will be impossible due to the inevitable death of neutrophils during sample storage. Similarly, we may not be able to obtain reference expression profiles for each cell type in targeted tissues. More importantly, reference gene signatures are often perturbed by the microenvironment or different treatment conditions^14^. Also, known marker genes may not be available for cell types that are not well-studied.

To the best of our knowledge, there is no existing deconvolution method that can employ partially known marker information to identify novel markers for cell types with/without initially known markers for deconvolution. To fill this gap, we propose a semi-supervised deconvolution method, semi-CAM. The proposed method is a two-stage procedure. In Stage I, we extend the unsupervised Convex Analysis of Mixtures (CAM) method^10^ to a partial marker-guided process to identify novel markers for all cell types; in Stage II, we impose a marker-based NMF method^19^ (denote it as semi-NMF) to estimate cell proportions using the identified markers information from stage I. Simulations and two real benchmark data are analyzed to compare semi-CAM with the unsupervised deconvolution CAM method, and the marker-based supervised deconvolution methods semi-NMF and DSA. The study also investigates the performance of semi-CAM from various numbers of known markers and numbers of cell types with known marker genes. We also apply semi-CAM to deconvolute blood cell proportions on Human Chlamydia-infection data.

## Methods

### Deconvolution model

Let ${{\rm{y}}}_{ij}$ be the observed bulk expression value for gene (or probe) $i$ and sample $j$, $i=1,\ldots ,m;j=1,\ldots ,n$. Given *K* cell types we model the observed bulk gene expression profile as:

1 $${y}_{ij}=\mathop{\sum }\limits_{k=1}^{K}{x}_{ik}{p}_{kj}+{e}_{ij},{e}_{ij} \sim N(0,{\sigma }^{2}),$$

where ${x}_{ik}$ is the gene expression level of gene $i$ with respect to cell type $k$; ${p}_{kj}$ is the proportion of cell type $k$ in sample $j$, $\,k=1,2,\ldots ,K$. We assume that ${x}_{ik}\ge 0$, ${p}_{kj}\ge 0$, and ${\sum }_{k=1}^{K}{p}_{kj}=1.$ Let $\,{Y}_{i}=({y}_{i1},\ldots ,{y}_{in}),$ ${X}_{i}=({x}_{i1},\ldots ,{x}_{iK})$, ${P}_{k}=({p}_{k1},\ldots ,{p}_{kn})$, ${E}_{i}=({e}_{i1},\ldots ,{e}_{in});$ and $Y=({Y}_{1},\ldots ,{Y}_{m}){\prime}$, $X=({X}_{1},\ldots ,{X}_{m}){\prime}$, $P=({P}_{1},\ldots ,{P}_{K}){\prime}$, $E=({E}_{1},\ldots ,{E}_{m}){\prime}$, Eq. (1) can be expressed in the matrix format:

2 $$Y=XP+E.$$

We assume that we have marker genes available on $L(\le K)$ cell types, where ${N}_{l}\,$is the number of known marker genes and ${M}_{l}=\{{M}_{l,1},\ldots ,{M}_{l,{N}_{l}}\}$ is the marker gene list for the *l*th $(l=1,\ldots ,L)$ cell type.

We propose the following two-stage semi-CAM method to estimate the cell type-specific expression and cell proportions.

### Stage I: identify novel marker genes for all cell types given partial marker genes

Similar to CAM, to identify marker genes for each cell type, we first employ k-means clustering to aggregate genes that have similar expression patterns into *C* clusters, and incorporate known marker genes in the clustering procedure (*C* is defined by the user). For *L* cell types with known marker genes, we form *L* initial cluster centers ${Z}_{1},\ldots ,{Z}_{L}$ by using averaged expressions of ${M}_{1},\ldots ,{M}_{L}$ marker genes, where ${Z}_{k}=\frac{1}{|i\in {M}_{k}|}{\sum }_{i\in {M}_{k}}{Y}_{i}$. The rest of the $C-L$ initial cluster centers ${Z}_{L+1},\ldots ,{Z}_{C}$ are randomly selected from the remaining genes (genes are not initially known markers) where ${Z}_{k{\prime} }\,={Y}_{i{\prime} }(k{\prime} =L+1,\ldots ,C,\,i{\prime} \notin {M}_{1}\cup {M}_{2},\ldots ,\cup {M}_{L})$. In the clustering procedure, we force that known marker genes for each cell type are always clustered together in one cluster by changing the expressions of those marker genes to their averaged expressions. We apply k-means clustering with these initial cluster centers $({Z}_{1},\ldots ,{Z}_{C})$, returning *C* clusters of similar genes with new cluster centers $({g}_{1},\ldots ,{g}_{C})$,

Assuming that the gene expression of a pure cell type is non-negative, cell type proportions are linearly independent, and cell-specific marker genes exist for each cell type in the bulk expression data, CAM finds marker genes by determining cluster centers as the optimal vertices of the convex hull constructed by $({g}_{1},\ldots ,{g}_{C})$. Given known marker genes from *L* cell types, i.e. ${M}_{1},\ldots ,{M}_{L}$, we extend CAM to a semi-supervised marker identification procedure. We treat $({g}_{1},\ldots ,{g}_{L})$ as part of the optimal vertices because they are the centers of the clusters of the known markers, and will identify the rest $K-L\,$optimal vertices from $C-L$ cluster centers through a total of ${C}_{C-L}^{K-L}$ searches. For each possible vertices set, margin-of-error (3)^10^ is used to calculate the distance between the vertices on the convex hull $({g}_{1},\ldots ,{g}_{L},{g}_{L+1},\ldots ,{g}_{K})$ and any other cluster centers ${g}_{c}\,(c\notin \,\{1,\ldots ,L,L+1,\ldots ,K\}).$

3 $${\delta }_{c}=\mathop{\min }\limits_{{\alpha }_{1},\ldots ,{\alpha }_{K}}||{g}_{c}-\mathop{\sum }\limits_{k=1}^{K}{\alpha }_{k}{g}_{k}|{|}_{2},{\alpha }_{k}\ge 0,\mathop{\sum }\limits_{k=1}^{K}{\alpha }_{k}=1.$$

$K-L$ optimal vertices are determined when the sum of the margin-of-error (4) reaches its minimum:

4 $$\{{g}_{1},\ldots ,{g}_{L},{g}_{{(L+1)}^{\ast }},\ldots ,{g}_{{K}^{\ast }}\}=argmi{n}_{\{1,\ldots ,K\}\in {C}_{C}^{K}}\mathop{\sum }\limits_{c=1}^{C}{\delta }_{c},$$

where $\{{(L+1)}^{\ast },\ldots ,{K}^{\ast }\}$ are the newly determined vertices. Therefore, $\{{g}_{1},\ldots ,{g}_{L},{g}_{{(L+1)}^{\ast }},\ldots ,{g}_{{K}^{\ast }}\}$ are the finally determined optimal vertices, $and\,{M}_{k}^{\ast }\,(k=1,\ldots ,L,{(L+1)}^{\ast },\ldots ,{K}^{\ast })$ are the identified novel marker genes for *K* cell types.

### Stage II: Estimate cell proportions

Once Stage I identifies a set of marker genes for each cell type, we utilize the marker-based Nonnegative Matrix Factorization (semi-NMF)^19^ to estimate cell proportions. The semi-NMF algorithm is based on the Nonnegative Matrix Factorization (NMF) algorithm^21^. Given a nonnegative observed mixed expression matrix *Y* of all the genes, the algorithm estimates cell type-specific expression matrix *X* of all the genes and mixing proportions matrix *P* that approximate $Y\approx XP$ by minimizing the function in (5) using iterative optimization.

5 $$\mathop{\min }\limits_{R(X,P)}D(Y,XP),$$

where *D* is the objective function describing the distance between *Y* and $XP,\,and\,R$ contains constraints on *X* and *P*.

The distance *D* used by us is Kullback-Leibler divergence^21^. The constraints imposed in *R* are: “non-negative” and “sum-to-one” on *P*. To incorporate the marker genes information into *X*, the elements of every row in *X* that corresponds to one of the marker genes will all be forced to zero, except one corresponding to the cell type of the marker gene. By doing so, the semi-NMF method efficiently incorporates the marker gene information, leading to improved estimation accuracy on *X* and *P*, as well as improved algorithm convergency compared with the unsupervised NMF method.

### Simulation

To assess the performance of semi-CAM, we generate cell type-specific expression data from benchmark immune cell line data GSE11058 by averaging the expressions of triplicated pure samples of each cell type. Then 10 mixing proportions are simulated from $U(0,1)$ and are scaled on each sample to assure that the proportions across all cells sum to one. The observed mixture expression of ${y}_{ij}$ is generated from (1) with ${e}_{ij}$ drawn from a normal distribution with standard deviation $\sigma ={2}^{\alpha {\log }_{2}{\sum }_{k=1}^{K}{x}_{ik}{p}_{kj}}.$ We vary $\alpha =0.85$ and 0.9 to control the magnitude of mixing noises. 100 data are simulated for each scenario.

## Results

### Simulation and real data analysis of immune cell benchmark data GSE11058

We conducted simulation analysis leveraging the pure cell line profiles from benchmark data GSE11058^22^ with varied simulated cell proportions to generate mixed expression profiles with normally distributed errors added. We also performed real data analysis using experimentally measured mixed expression profiles and cell line proportions from GSE11058. To investigate the impacts of markers information on the cell proportion estimations by semi-CAM method, we compare the proposed semi-CAM to three alternative methods: (1) the unsupervised deconvolution method CAM^10^, which does not require any prior information and only takes observed mixture expressions as input data; (2) when marker genes for each cell type are available, the two supervised marker-based deconvolution methods semi-NMF and DSA^18^ are assessed. The DSA method only uses known marker genes for cell proportion estimations, while the semi-NMF method forces block-like expression patterns for marker genes, but treats the other genes as unconstrained and adopts all genes for cell proportion estimations.

The benchmark data GSE11058 contains four immune cell types, Jurkat, IM-9, Raji, and THP-1. The four immune cell lines were grown and run on microarrays either by themselves or in mixtures of various relative proportions. Each pure cell sample has three technical replicates, resulting in 12 total pure samples; 4 different mixture proportions were generated to create 12 bulk samples in triplicate for each mixture proportion. The data were analyzed with Microarray Suite version 5.0 (MAS 5.0) using Affymetrix default analysis settings and global scaling as the normalization method. The trimmed mean target intensity of each array was arbitrarily set to 500. The normalized data were downloaded from the NCBI GEO database^4^.

**Figure 1:**
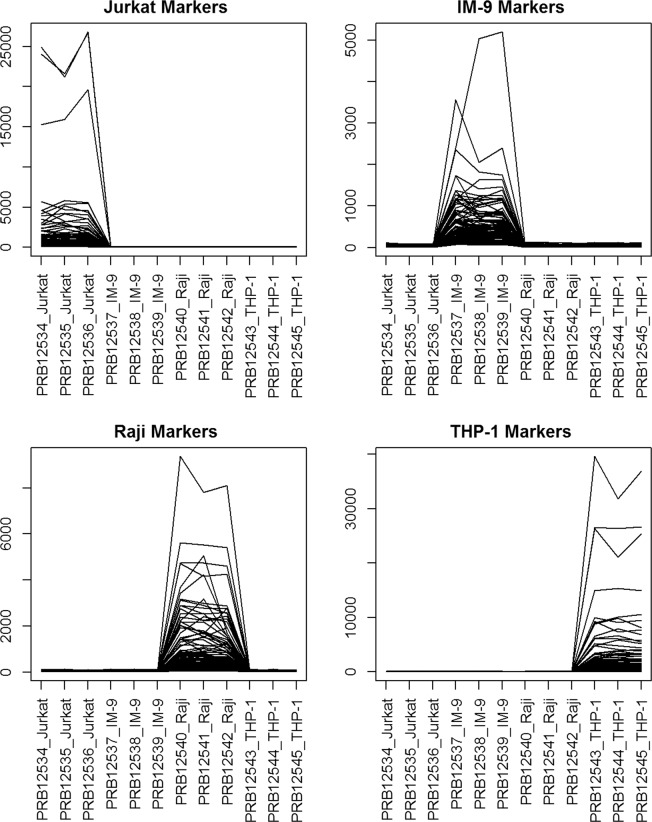
Expressions of marker genes for Jurkat, IM-9, Raji, and THP-1 cells from the immune cell line data GSE11058. X-axis: pure cells; Y-axis: expression levels of marker genes. The selected marker genes highly express on one cell and barely express on other cells, suggesting they are ideal markers.

We directly identify marker genes from pure cell expression by performing a standard t-test comparing the expressions of the highest expressed cell type and second highest expressed cell type^22^. The p-values are calculated on the log2 transformed data, using a two-sided t-test with equal variance. We identify markers as the probesets with a p-value less than 0.05, log2 fold change greater than 1.5, and the maximum expression of the second highest expressed cell type less than 2^7^. Only the top 100 markers with the smallest p-values for each cell type are selected, and the corresponding FDRs are summarized in Table 1. The expression levels of the identified markers for Jurkat, IM-9, Raji, and THP-1 cells are plotted in Fig. 1. It is clear that the selected marker genes are highly expressed on one cell type but barely expressed on the other cell types, suggesting they are ideal markers. When conducting semi-supervised or supervised deconvolution methods with these marker genes, we alter the percentage of marker genes for each cell type being 10%, 50%, and 100%. Also, we vary the number of cell types with known marker genes $L=1,2,3,4$ for simulation and real benchmark data. The averaged correlations of the semi-CAM method utilizing known markers of all possible combinations of *L* cell types are calculated as the results of the semi-CAM method with maker genes for *L* cell types.

**Table 1:** The FDRs of the top 100 marker genes for Jukat, IM-9, Raji, and THP-1 cells from the cell line data GSE11058.

	Cell type			
	Jukat	IM-9	Raji	THP-1
FDR	0.014	0.032	0.039	0.007

**Figure 2:**
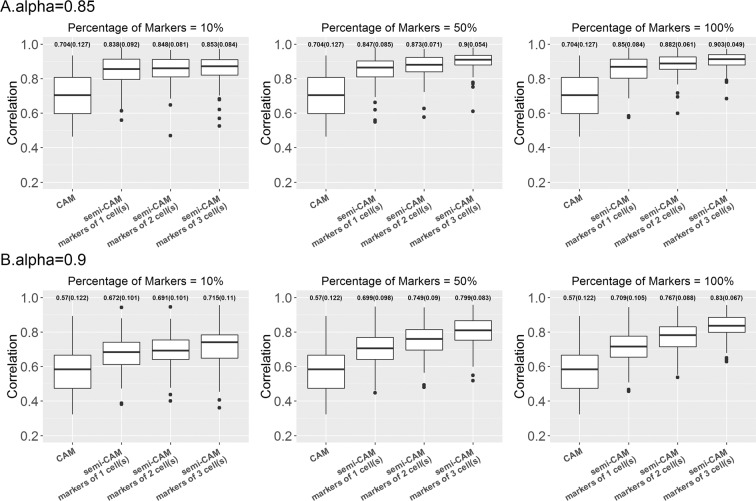
The proposed semi-CAM method outperforms the unsupervised CAM method in simulated data with weak (2A) and strong (2B) noise, when known marker genes for partial cell types are available. X-axis indicate methods CAM and semi-CAM with initially known marker genes for 1,2,3 cell(s). Y-axis shows the Pearson correlations between the estimated and true proportions. The averaged correlations (standard deviations of correlations) is presented on the top of each boxplot. 10%, 50%, and 100% of marker genes are used for the semi-CAM method.

**Figure 3:**
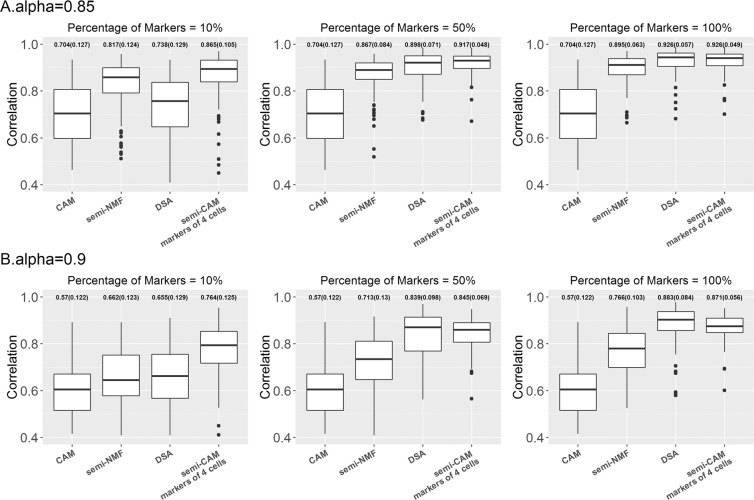
The proposed semi-CAM method outperforms the unsupervised CAM method and the supervised DSA and semi-NMF methods in simulated data with weak (2A) and strong (2B) noise, when known marker genes for all four cell types are available. X-axis indicate the CAM, semi-NMF, DSA and semi-CAM methods, with initially known marker genes for all four cell types. Y-axis shows the Pearson correlations between the estimated and true proportions. The averaged correlations (standard deviations of correlations) are presented on the top of each boxplot. 10%, 50%, and 100% of marker genes for each cell type are used for semi-NMF, DSA and semi-CAM.

**Figure 4:**
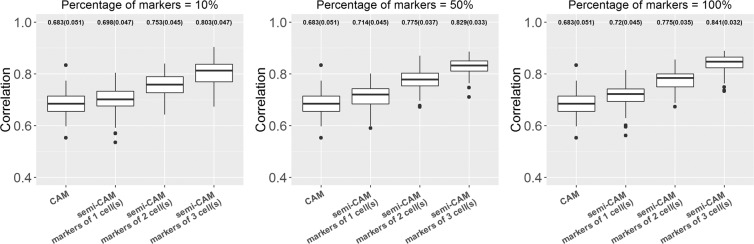
The proposed semi-CAM method outperforms the unsupervised CAM method in real data analysis of benchmark cell line data GSE11058, when the known marker genes for partial cell types are available. X-axis indicates the CAM and semi-CAM methods with initially known marker genes for 1, 2, or 3 cell type(s). Y-axis shows the Pearson correlations between the estimated and true proportions. The averaged correlations (standard deviations of correlations) are presented on the top of each boxplot. 10%, 50%, and 100% of marker genes are used for the semi-CAM method.

**Figure 5:**
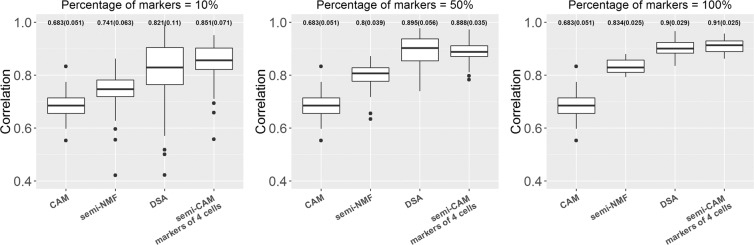
The proposed semi-CAM method outperforms the unsupervised CAM method and the supervised DSA and semi-NMF methods in real data analysis of benchmark cell line data GSE11058, when the known marker genes for all four cell types are available. X-axis indicates the CAM, semi-NMF, DSA and semi-CAM methods with the initially known marker genes for all four cell types. Y-axis shows the Pearson correlations between the estimated and true proportions. The averaged correlations (standard deviations of correlations) is presented on the top of each boxplot.10%, 50%, and 100% of marker genes for each cell type are used for semi-NMF, DSA and semi-CAM.

For simulation and real data analyses, we first filter 50% of the genes with low expression levels. Second, we only keep 50% of those genes with the top coefficient of variance (CV). After deconvolution, we map the estimated proportions to actual cell types in the mixtures and use the Pearson correlations between estimated and true cell proportions to evaluate the performance of the methods. The results from 100 simulations are presented in Figs. 2 and 3. For the real benchmark data, we randomly choose one sample from three replicated mixture samples to form one data on which deconvolution analyses are performed. We repeat the process 100 times; the summarized results are presented in Figs. 4 and 5.

When marker genes for $L(\le K)$ cell types are known, we compare the performance of the unsupervised method CAM and the semi-supervised method semi-CAM. The results of the simulation data are presented in Fig. 2 and the results of the real data GSE11058 are in Fig. 4. Simulation and real benchmark data analyses show consistent results. By only utilizing 10% of markers from even one cell, semi-CAM enhances the cell proportion estimations over CAM. Generally, increasing the number of cells with known markers or/and the number of markers per cell improves the performance of semi-CAM. This demonstrates the advantage of incorporating partial known marker information into deconvolution.

When markers for all four cells are available, CAM, semi-CAM and two other supervised marker-based methods (semi-NMF and DSA) can be compared. Generally, semi-CAM achieves more precise proportion estimations than semi-NMF. The mean correlations of semi-CAM are similar to DSA when the number of known markers for each cell are relatively large (for example, 50% or 100% markers per cell), but semi-CAM has lower standard deviations. The improvement is because semi-CAM searches for novel markers besides the initially known markers used by semi-NMF and DSA. In summary, when partially known markers for partial cells are given, semi-CAM outperforms CAM; when markers for all cells are available, semi-CAM performs in a manner superior or comparable to the semi-NMF and DSA methods.

**Figure 6:**
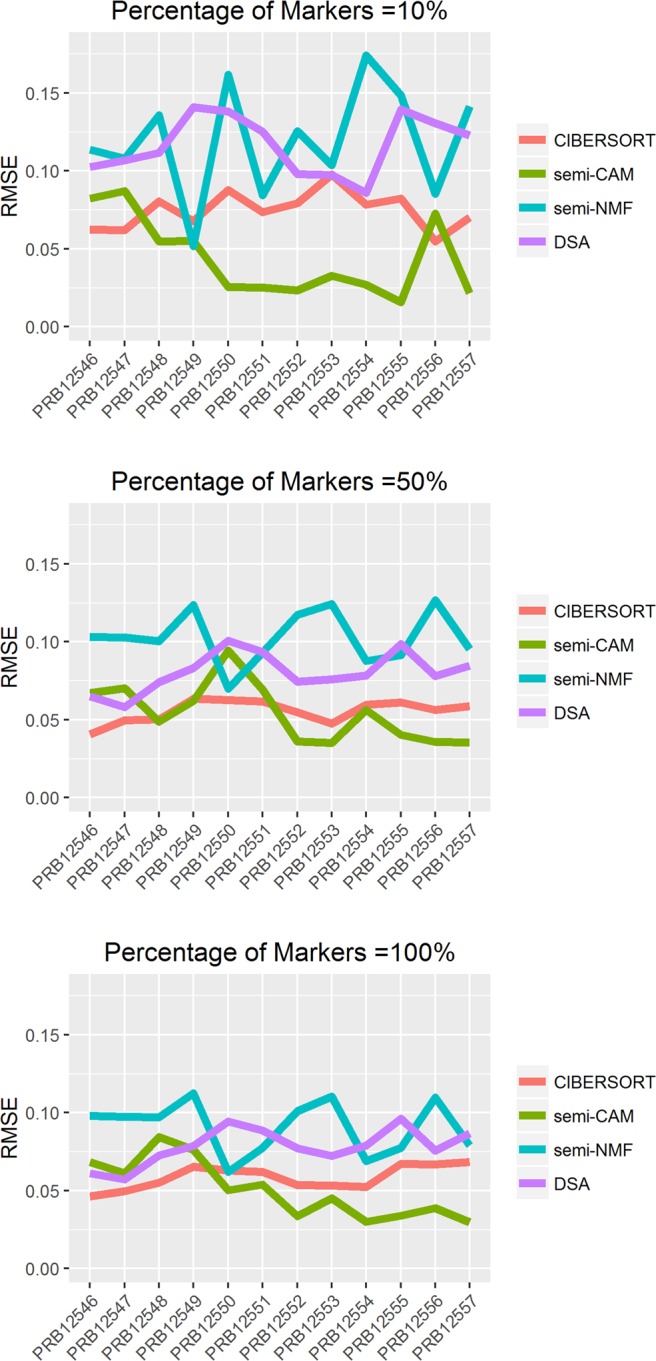
Deconvolution immune cell line data GSE11058 with varied percentage of known markers. X-axis indicates the mixed sample ID. Y-axis shows root-mean-square error (RMSE). We assess the performance of CIBERSORT, semi-CAM, semi-NMF, and DSA methods using 10%, 50%, 100% of marker genes respectively.

We then compared the proposed method with CIBERSORT, semi-NMF and DSA on the benchmark data GSE11058 with all 12 bulk samples, using the signature genes provided by CIBERSORT as the initial marker genes. The results from CIBERSORT, semi-CAM, semi-NMF and DSA with the use of 10%, 50%, 100% of markers are shown in Fig. 6 where root-mean-square error (RMSE) are calculated per mixture. The results show that generally the proposed method achieves smaller RMSE among the methods, especially when only using 10% of markers.

### Real data analysis of female mixed breast and blood tissues benchmark data GSE29832

The benchmark data GSE29832^12^ contains profiles of pure/mixed blood and breast tissues from female adults. Each pure blood and breast tissue sample had 3 replicates, which were mixed in two different proportions to generate 9 mixed samples^12^. The RMA normalized data were downloaded from the NCBI GEO database^4^.

**Figure 7:**
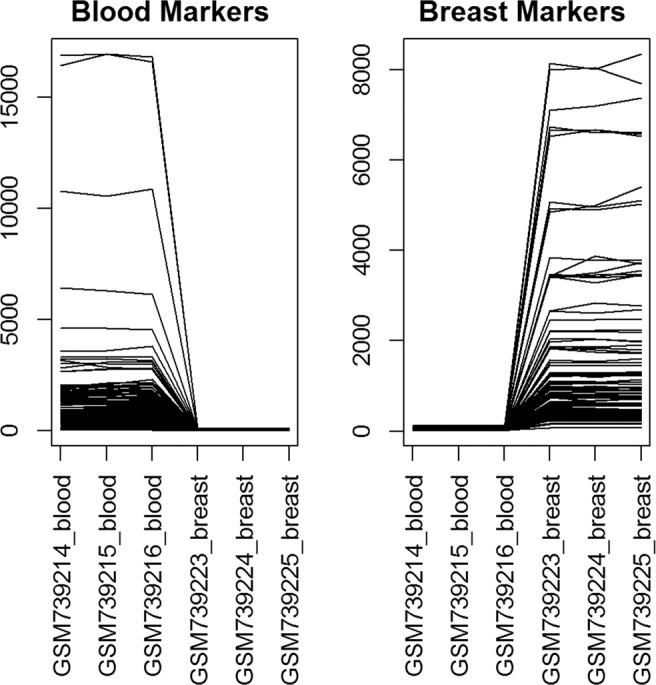
Expressions of marker genes for blood and breast from the mixed breast and blood tissues benchmark data GSE29832. X-axis: pure tissues; Y-axis: expression levels of marker genes. The selected marker genes highly express on one cell and barely express on other cells, suggesting they are ideal markers.

**Figure 8:**
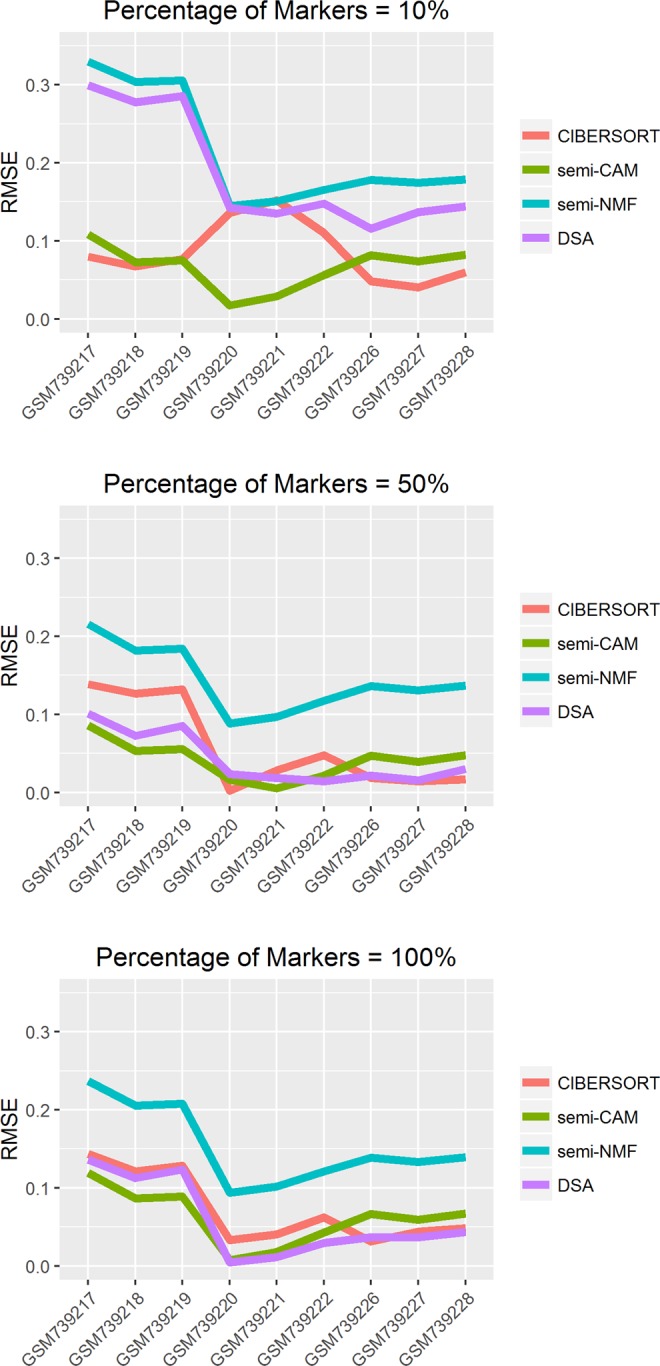
Deconvolution of real benchmark tissue data GSE29832 with varied percentage of known markers. X-axis indicates the mixed sample ID. Y-axis shows root-mean-square error (RMSE). We assess the performance of CIBERSORT, semi-CAM, semi-NMF, and DSA methods using 10%, 50%, 100% of marker genes respectively.

We apply the same data filtering procedure as the benchmark data GSE11058. The same marker identification criteria, as described in analysis of immune cell benchmark data, are used on the 6 pure samples to identify the top 100 markers for each tissue. The expression levels of the identified markers for blood and breast are plotted in Fig. 7. We calculate root-mean-square error (RMSE) as evaluation metric to compare semi-CAM with CIBERSORT, DSA and semi-NMF and the results are summarized in Fig. 8. When only 10% of markers are provided as the initial markers, semi-CAM outperforms DSA and semi-NMF dramatically; compared with CIBERSORT, it achieves lower RMSE for some samples. When the number of initial markers increase to 50% or 100%, semi-CAM performs much better than semi-NMF, and comparable with CIBERSORT and DSA.

### Real data analysis of human chlamydia-infection dataset

*Chlamydia trachomatis* is the leading bacterial sexually transmitted infection and can cause severe female reproductive morbidities. Our prior studies using bulk blood mRNA gene expression network analysis revealed that different responses in chlamydial-infected women with infection limited to the cervix versus those with endometrial infection and disease^23,24^. These data indicate that *chlamydia trachomatis* drives differential responses that can be detected in the peripheral blood.

We will leverage data obtained from 37 asymptomatic Chlamydia infected women who participated in a previously described T cell Response Against Chlamydia (TRAC) cohort^25^ and were followed up for one year. These 37 women (age 15–30 years), who were infected at enrollment, were assigned to two groups after the one year follow up: women who subsequently uninfected were defined as protected (n = 13), while those women who became infected were defined as susceptible (n = 24). Blood bulk gene expression was profiled by gene expression arrays (Illumina HT-12 v4 Expression BeadChips)^23,24^ and cell counts except for neutrophils were measured by mass cytometry. Counts of 5 cell types in the blood, including B cells, T cells, NK cells, monocytes, and non-NK non-monocyte cells, were used in this study to evaluate the performance of deconvolution methods. The Institutional Review Boards for Human Subject Research at the University of Pittsburgh and the University of North Carolina approved the study and all participants provided written informed consent prior to inclusion.

**Figure 9:**
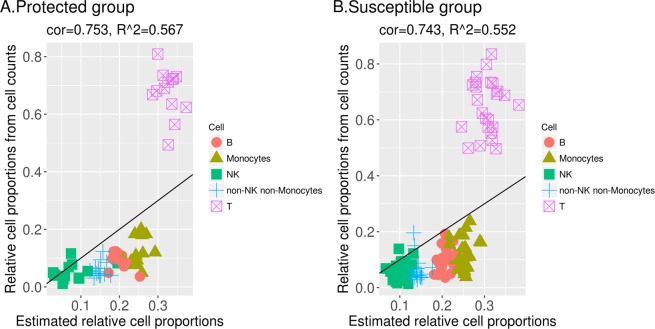
CAM estimated relative cell proportions and relative cell proportions obtained from cell counts of B cells, T cells, NK cells, monocytes, and non-NK non-monocytes in the protected group (**A**) and the susceptible group (**B**).

**Figure 10:**
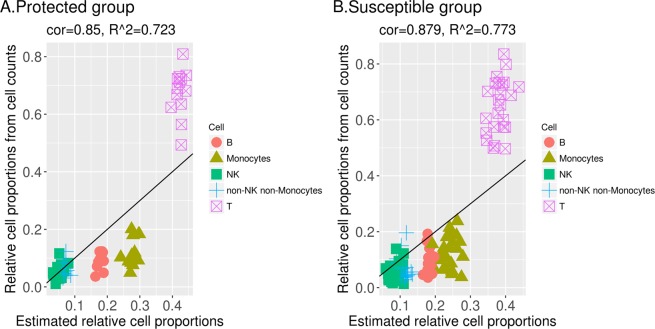
Semi-CAM estimated relative cell proportions and relative cell proportions obtained from cell counts of B cells, T cells, NK cells, monocytes, and non-NK non-monocytes in the protected group (**A**) and susceptible group (**B**).

We apply CAM and semi-CAM methods to deconvolute the bulk gene expression data from two groups, protected and susceptible women, separately. The data are first preprocessed by filtering 50% low expression genes and low variation genes. Since both methods have some randomness during the clustering procedure, for each approach we deconvolute 100 times with different seeds and take the average proportions as the final cell proportion estimation. We choose gene FUT4 as the marker gene for neutrophils, CD19 for B cells, and CD3E for T cells when applying the semi-CAM method. The estimated relative cell proportions and relative cell proportions calculated from cell counts of B cells, T cells, NK cells, monocytes, and non-NK non-monocytes are shown in Figs. 9 and 10. For the CAM method, the correlation between the estimated and calculated relative cell proportions is 0.753 (R-squared=0.567) for the protected group and 0.743 (R-squared=0.552) for the susceptible group. For the semi-CAM method, the correlation between the estimated and calculated relative cell proportions increases to 0.85 (R-squared=0.723) for the protected group and 0.879 (R-squared=0.773) for the susceptible group. The deconvolution results suggest that semi-CAM obtains more accurate cell proportions than CAM. Thus, semi-CAM enables us to determine differences in cellular responses among subgroups of women with differing degrees of infection and levels of susceptibility to reinfection.

## Discussion

Deconvolution bulk transcriptomic data to cell type proportion and cell type-specific expression are essential to understand the cell type-specific responses to disease mechanisms. The existing bulk transcriptomic data deconvolution methods are either unsupervised methods without incorporating any cell type-specific information, or supervised methods requiring cell proportion, reference gene signatures, or marker genes for each cell type. To fully utilize partial cell-specific information, we propose a semi-supervised marker-guided deconvolution method, semi-CAM, to adopt partially known marker gene information in the deconvolution procedure. Simulation data and real benchmark microarray data are used to validate the proposed method. The results demonstrate that semi-CAM achieves more precise cell proportion estimations than the unsupervised deconvolution method CAM by incorporating marker genes for partial tissues/cells. When markers for all tissues/cells are available, semi-CAM approaches or even exceeds the performance of the supervised method using signature genes, CIBERSORT, as well as the marker-based methods semi-NMF and DSA. We assess semi-NMF and DSA methods for cell proportion estimations (Fig. S1) and utilize semi-NMF in stage II of semi-CAM due to its superior performance on the stage I identified markers. semi-NMF algorithm has an option of using Kullback-Leibler divergence or Euclidean distance. In our analysis, Kullback-Leibler divergence is selected because we observe its consistently better deconvolution results than those from the regular Euclidean distance from our extensive simulation and real data analysis (Fig. S2). Besides using perfect cell type-specific marker genes for deconvolution, we also demonstrate the advantages of semi-CAM using imperfect marker genes (Fig. S3–S4). In addition, we show the accuracy of semi-CAM by evaluating the method using averaged correlation and square root of mean square error (RMSE) (Fig. S5–S6). An application of a *Chlamydia-infection* data set also indicates the semi-CAM method outperforms CAM in blood cell proportion estimations. In conclusion, the proposed method semi-CAM successfully imposes partially known marker gene information for bulk data deconvolution, which would be very useful in practical problems where only marker genes for partial cell types are available. A potential question for unsupervised deconvolution is how to map the deconvoluted results to specific cell types without other reference information. One solution is to use the pathway analysis suggested by CAM, where the deconvoluted cell type-specific gene signatures can be incorporated into Ingenuity Pathway Analysis (IPA)^26^ (QIAGEN Inc., https://www.qiagenbioinformatics.com/products/ingenuitypathway-analysis), a database of gene annotations and biological functions and used to search for the known biological function of each marker set. We suggested the same strategy for semi-CAM.

One limitation of the current deconvolution methods, including semi-CAM, is that confounding covariates cannot be adjusted in models. Since most deconvolution methods assume non-negative for the expression profiles, residuals from regression cannot be used to control for the confounding factors before deconvolution. Future research is warranted to address this issue.

## Supplementary information

Supplementary Information.

## Data Availability

The microarray human chlamydia-infection data are in GEO (https://www.ncbi.nlm.nih.gov/geo/query/acc.cgi?acc=GSE110106) and the cell proportion data will be submitted for publication in an original biological paper soon. Public gene expression data analyzed in this paper are also available from the Gene Expression Omnibus Database under Accession Number GEO: GSE11058, GSE29832. Other datasets generated and analyzed during the current study are available from the corresponding author on reasonable request. The semi-CAM codes for deconvolution are available at https://github.com/ylidong/semi-CAM.
