# Supplementary material for: Semi-CAM: A semi-supervised deconvolution method for bulk transcriptomic data with partial marker gene information
Source: Sci Rep. 2020 Mar 25;10:5434. doi: 10.1038/s41598-020-62330-2 (PMC7096458; doi:10.1038/s41598-020-62330-2)
Supplement: Supplementary file 1 — Supplementary Information. [file 41598_2020_62330_MOESM1_ESM.docx]

**Semi-CAM: A semi-supervised deconvolution method for bulk transcriptomic data with partial marker gene information**

Li Dong^1^, Avinash Kollipara^2^, Toni Darville^2^, Fei Zou^1^*, Xiaojing Zheng^1,2^*

^1^Department of Biostatistics, ^2^Department of Pediatrics, University of North Carolina at Chapel Hill, Chapel Hill, North Carolina, USA.

**Supplementary Methods**

**semi-NMF and DSA for cell proportion estimations**

semi-CAM applies semi-NMF algorithm in the second step after identifying a set of cell type specific markers to estimate the cell proportions. semi-NMF uses all genes besides initial marker genes for the cell proportion estimates; however, DSA only uses initial marker genes instead. We evaluated the performances of DSA and semi-NMF with CAM identified marker genes (denoted as CAM.DSA, and CAM.NMF, respectively) as well as semi-CAM stage I identified marker genes (denoted as semi-CAM.DSA and semi-CAM.NMF, respectively). The results of all the methods on the real benchmark data are shown in Figure S1. The proposed method semi-CAM (which is essentially semi-CAM.NMF in the figure (yellow highlighted)) outperforms all the other methods.

**Evaluating the impact of imperfect markers**

To evaluate the performance of semi-CAM on some imperfect markers (markers which highly express on more than on cell types). We selected 80 imperfect markers for each cell type to conduct the analysis. The gene expressions of the marker genes are shown in Figure S3. The semi-CAM results with the initial markers from all four cell types are presented and evaluated by correlation, averaged correlation of four cell types, and the square root of mean square error (RMSE) of the estimated and true proportion matrix for the benchmark data in Figure S4. We compared our method semi-CAM with unsupervised method CAM and supervised methods semi-NMF and DSA. All evaluation metrics show that our method outperforms CAM, semi-NMF and DSA.

**Evaluate semi-CAM and CAM methods using averaged correlation of four cell types and square root of mean square error (RMSE).**

Besides Pearson correlation between estimated and true proportions, we used averaged correlation of four cell types, and the square root of mean square error (RMSE) of the estimated and true proportion matrix to assess the deconvolution performances on the benchmark data GSE11058. The results are shown in Figure S5 for using markers for 1,2,3 cell types; and in Figure S6 for using markers for all four cell types. It is clear that Pearson correlation, average of averaged Pearson correlation of four cell types and RMSE results are quite consistent, and that semi-CAM outperforms the CAM, semi-NMF and DSA.

**Supplementary Figures**


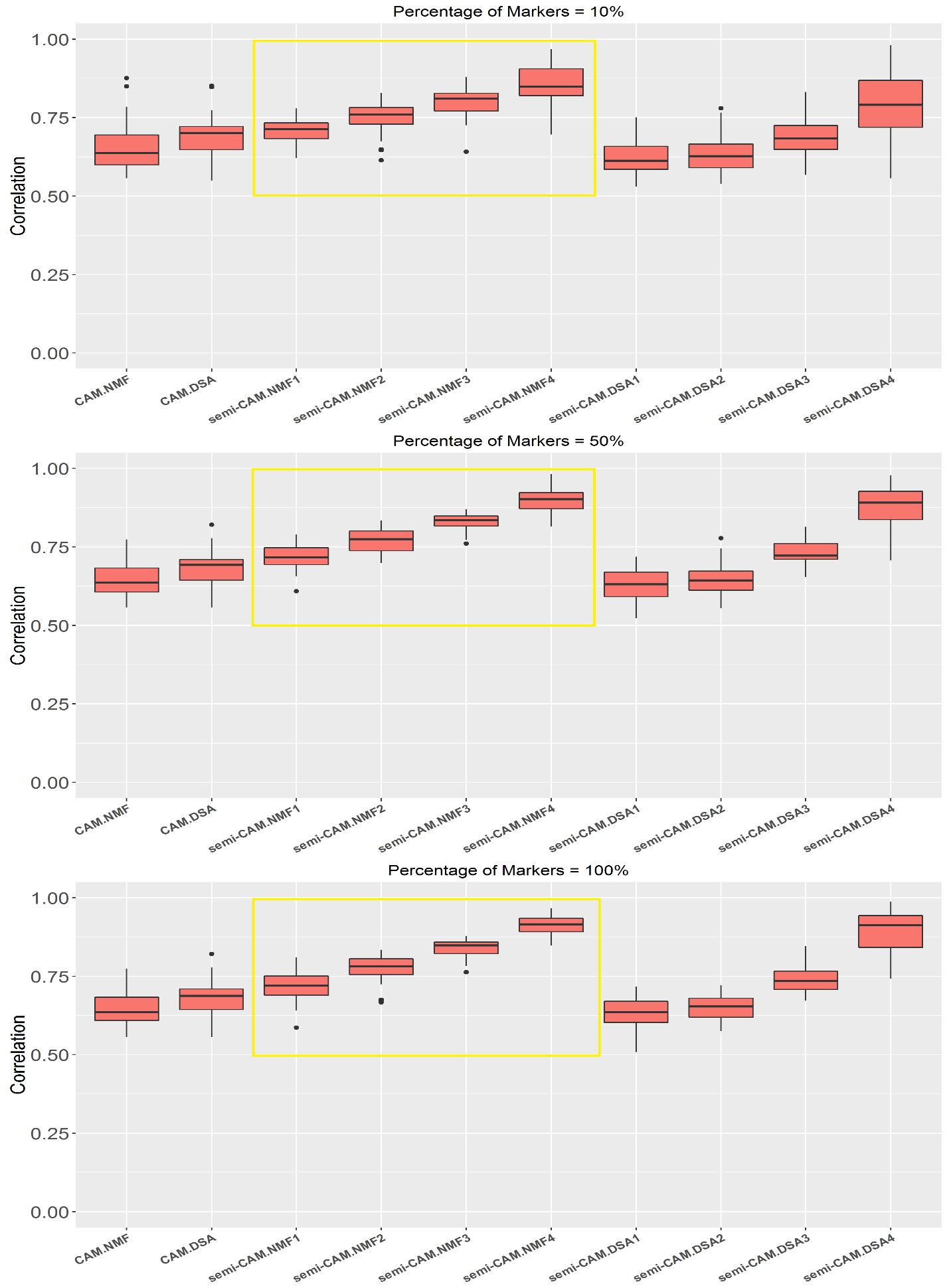
Figure S1. Comparisons of semi-NMF and DSA methods with CAM and semi-CAM stage I identified markers on estimating cell type proportions. DSA and semi-NMF with CAM identified marker genes are denoted as CAM.DSA, and CAM.NMF, respectively; with semi-CAM stage I identified marker genes are denoted as semi-CAM.DSA and semi-CAM.NMF, respectively. The proposed method semi-CAM (which is semi-CAM.NMF as referred in the figure (yellow highlighted)) outperforms all the other methods.


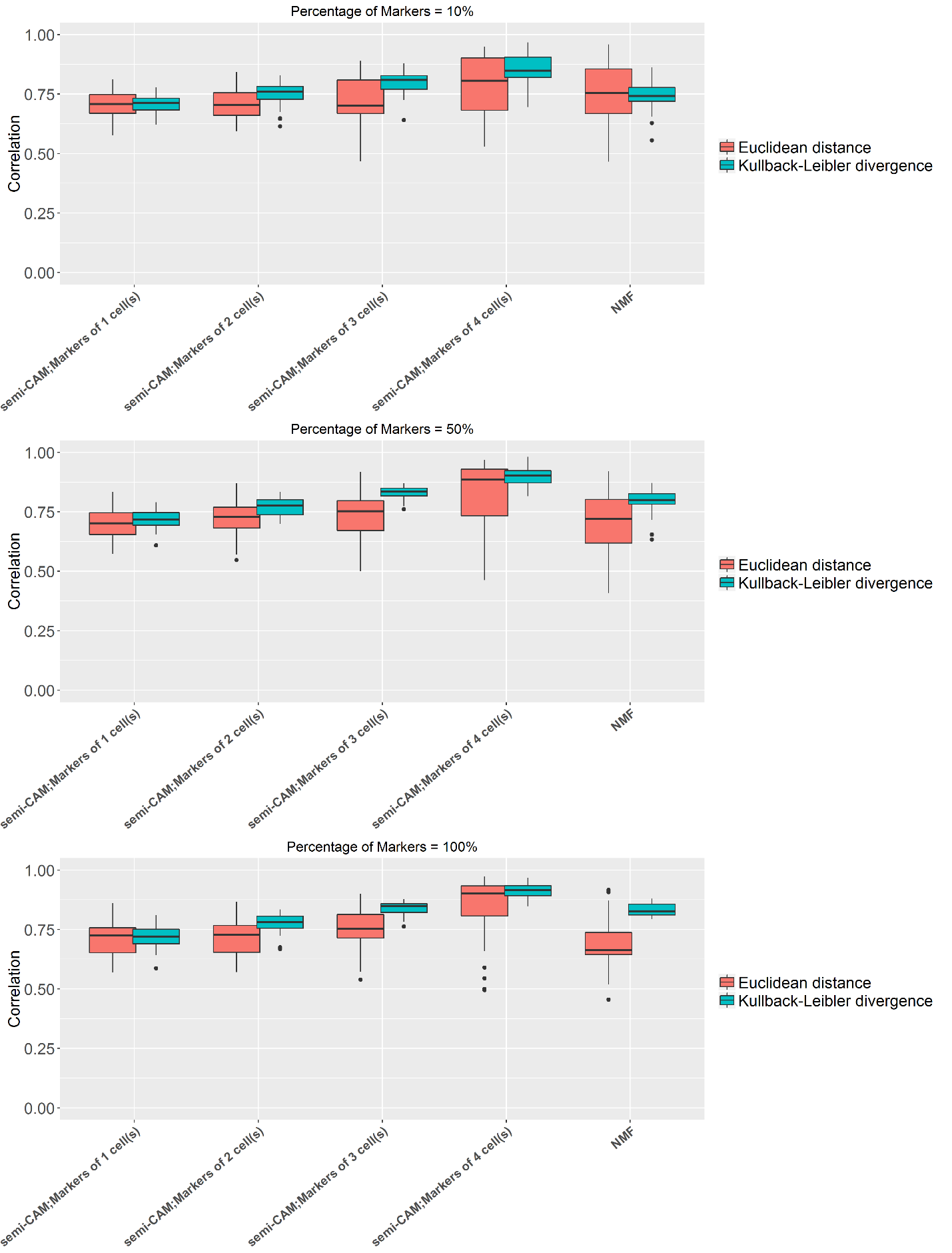


Figure S2. The analysis results of benchmark data GSE11058 from semi-CAM and NMF with the use of Kullback-Leibler divergence and Euclidean distance. Clearly Kullback-Leibler divergence outperforms Euclidean distance, especially when the percentage of markers used is high (greater than or equal to 50%).


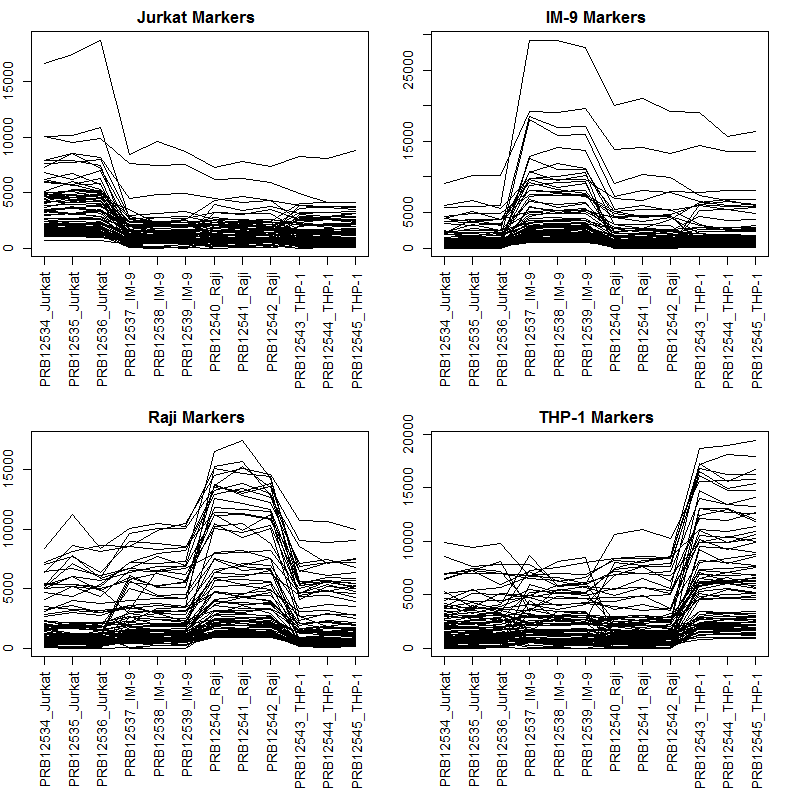


Figure S3. Expression profiles of the selected (imperfect) marker genes across the pure cell samples from the benchmark data GSE11058. X-axis: pure cells sample id; Y-axis: expression levels. The selected marker genes are clearly imperfect as some are highly expressed on more than one cell types.


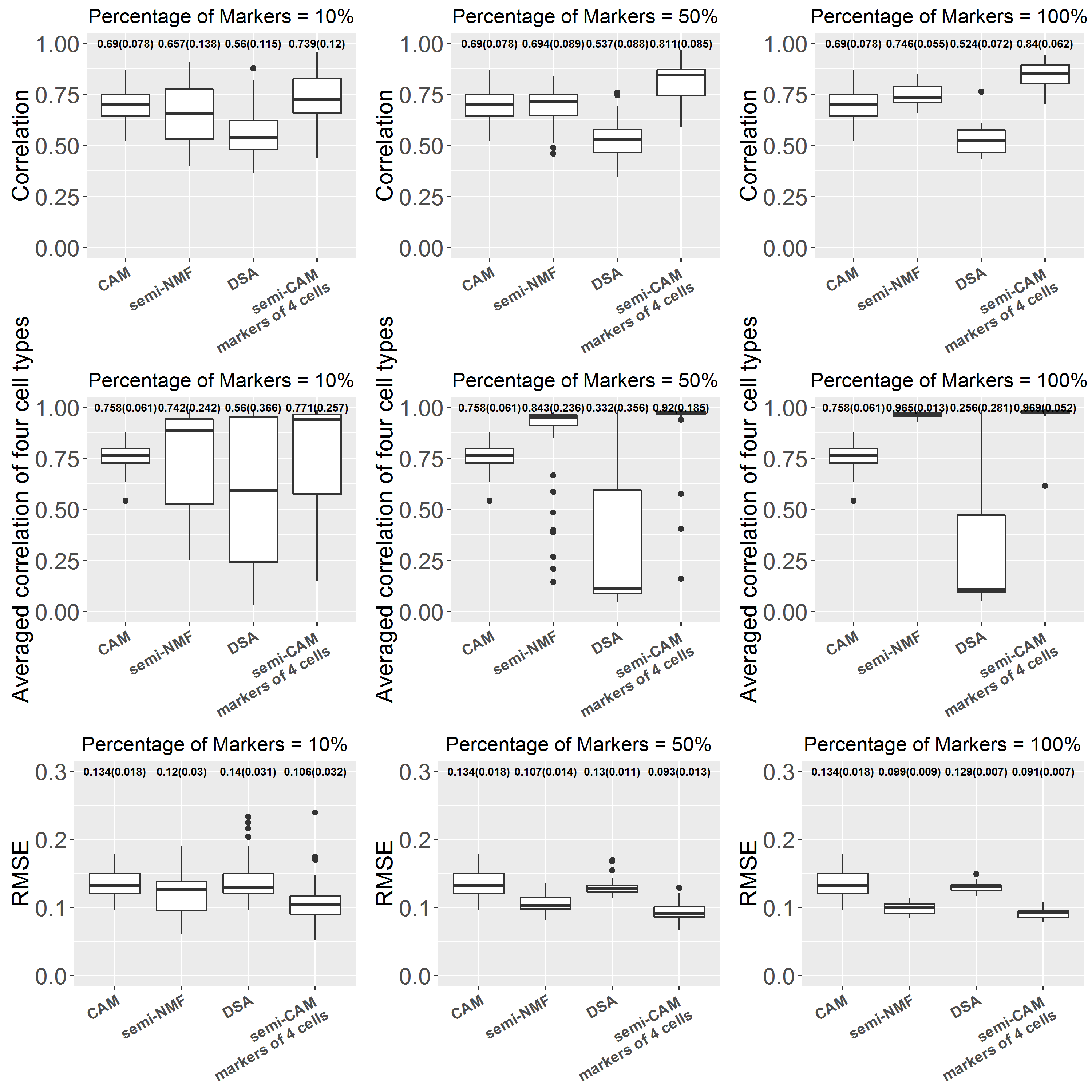


Figure S4. Evaluation the performances of CAM, semi-NMF, DSA and semi-CAM methods on the benchmark data GSE11058, when using the imperfect markers for all four cells. X-axis indicates the CAM, semi-NMF, DSA and semi-CAM methods. Y-axis shows the Pearson correlations between the estimated and true proportions, averaged Pearson correlation of four cell types and RMSE. The averaged correlations, average of averaged correlations and RMSE (standard deviations) are presented on the top of each boxplot.


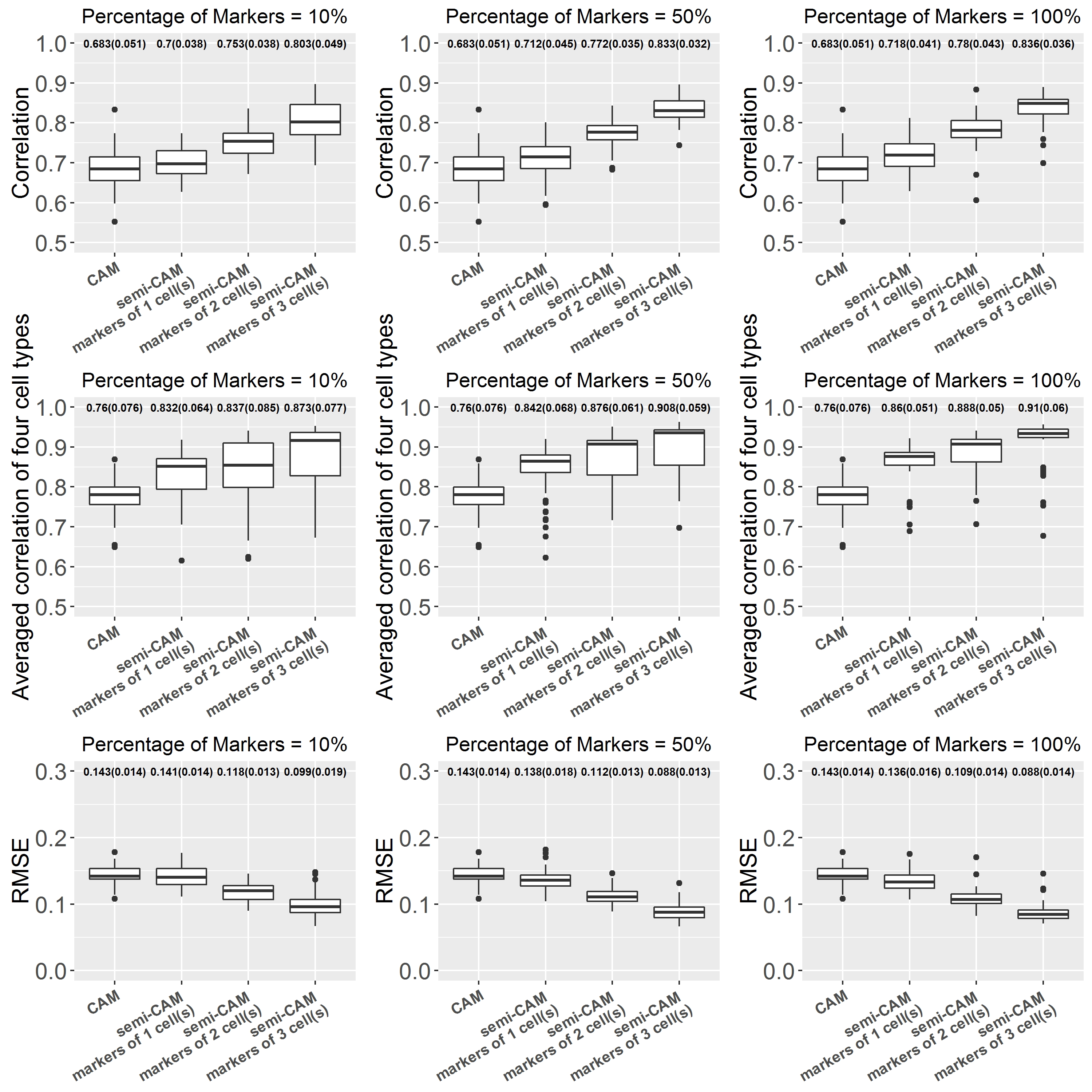


Figure S5. Evaluation the performances of CAM and semi-CAM on the benchmark immune cell line data GSE11058, when known marker genes for partial cell types are available. X-axis indicates the CAM and semi-CAM methods with initially known marker genes for 1,2, or 3 cell type(s). Y-axis shows the Pearson correlations between the estimated and true proportions, averaged Pearson correlation of four cell types and RMSE. The averaged correlations, average of averaged correlations and RMSE (standard deviations) are presented on the top of each boxplot. 10%, 50%, and 100% of marker genes are used for the semi-CAM method.


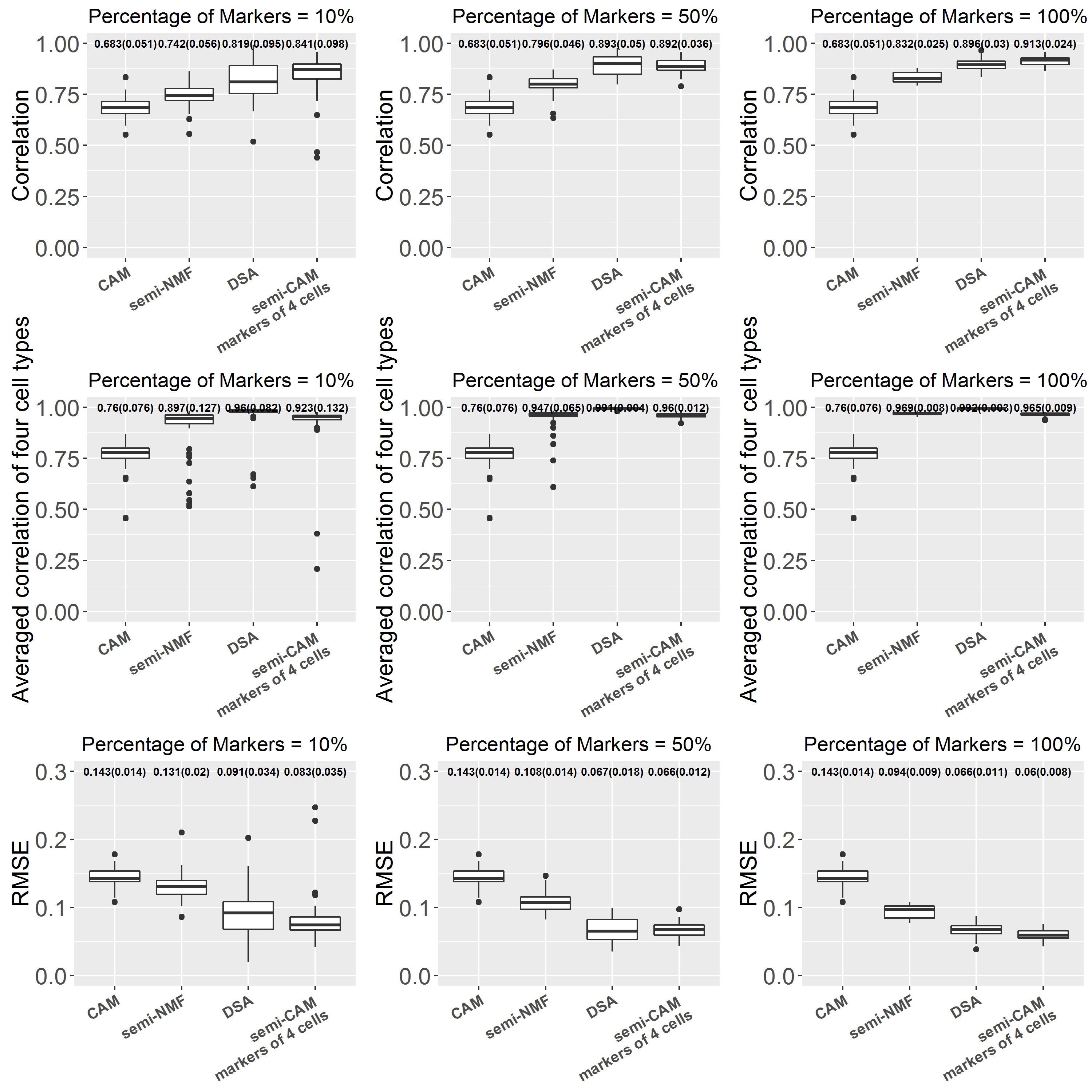


Figure S6. Evaluation the performances of CAM, semi-NMF, DSA and semi-CAM on the benchmark immune cell line data GSE11058 when the known marker genes for all four cell types are available. X-axis indicates the CAM, semi-NMF, DSA and semi-CAM methods with the initially known marker genes for all four cell types. Y-axis shows the Pearson correlations between the estimated and true proportions, averaged Pearson correlation of four cell types and RMSE. The averaged correlations, average of averaged correlations and RMSE (standard deviations) are presented on the top of each boxplot. 10%, 50%, and 100% of marker genes are used for the semi-CAM method.
